# Layered Double Hydroxide/Graphene Quantum Dots as a New Sorbent for the Dispersive Solid-Phase Microextraction of Selected Benzophenones, Phenols, and Parabens

**DOI:** 10.3390/molecules27238388

**Published:** 2022-12-01

**Authors:** Theodoros Chatzimitakos, Alkiviadis Vasilas, Constantine Stalikas

**Affiliations:** Laboratory of Analytical Chemistry, Department of Chemistry, University of Ioannina, 45110 Ioannina, Greece

**Keywords:** layered double hydroxides, graphene quantum dots, sample preparation, benzophenones, phenols, parabens, HPLC

## Abstract

In this study, the synthesis of a layered double hydroxide (LDH) composite with graphene quantum dots (GQDs) and its utilization for the development of a dispersive solid-phase extraction procedure are described. To this end, a carbonate-free Mg-Al LDH was synthesized. The development of the composite material made feasible the use of GQDs in a sample preparation procedure, while the incorporation of the GQDs in the LDH structure resulted in an 80% increase in extraction efficiency, compared to the bare LDH. As a proof of concept, the composite material was used for the development of an analytical method for the extraction, and preconcentration, of benzophenones, phenols, and parabens in lake water using high-performance liquid chromatography, coupled to a diode array detector. The analytical method exhibits low limits of quantification (0.10–1.33 μg L^−1^), good recoveries (92–100%), and satisfactory enrichment factors (169–186). Due to the abovementioned merits, the easy synthesis and simple extraction, the developed method can be used for the routine analysis of the target compounds.

## 1. Introduction

The use of nanomaterials, nowadays, in many different applications is increasing exponentially. Many of them are employed in sample preparation procedures in an effort to develop analytical methods with enhanced characteristics. For instance, carbon nanotubes have widely been used for the sorption of a wide variety of analytes, including pesticides, antibiotics, polycyclic aromatic hydrocarbons, estrogens, etc. [1,2] for sample preparation procedures.

Since their discovery in 2004 by Xu et al., graphene quantum dots (GQDs) have drawn a lot of attention due to their multiple applications [3]. These are widely spread through many fields, including biomedicine and optoelectronics, drug delivery, fluorescence imaging, nano-sensors, etc. [4,5]. This type of nanomaterial mounts multiple benefits, such as a small particle size and large surface area, inexpensive synthetic procedures, an environmentally friendly nature, a chemical stability, limited toxicity, high water dispersibility, and plenty of oxygen-containing functional groups (-OH, -C=O, and -COOH) on their surface [6,7,8]. These oxygen-containing groups enhance the sorption properties of the GQDs for organic pollutants, which can further be enhanced through surface functionalization [9,10]. Despite this property, GQDs have only recently started to be used as sorbent materials in sample preparation procedures [11,12]. Almost exclusively, they have been used in analytical chemistry to develop photoluminescent-based sensors. However, the use of GQDs as sorbents is highly promising and sets a new trend in analytical chemistry [13,14,15]. Despite their multiple advantages, their high water dispersibility makes it almost impractical to be used as sorbents in aqueous solutions. To overcome this hindrance, GQDs should be immobilized into a micro-carrier.

Layered double hydroxides (LDH) are brucite-like layered materials with a general formula of [$M_{1-x}^{2+}M_{x}^{3+}$(OH)_2_]^x+^[A^n−^]_x/n_∙mH_2_O [16], and they can be suitable supporting materials for the utilization of carbon dots. The trivalent- and the divalent-layer cations of the formula are represented as M^3+^ and M^2+^, respectively, and the interlayered charge-balancing anion as A^n−^. Common metal cations used for the LDH synthesis are the divalent Ni^2+^, Co^2+^, Mg^2+^, Mn^2+^, Cu^2+^, and Ca^2+^ and the trivalent Ga^3+^, Fe^3+^, Al^3+,^, and Cr^3+^. Common interlayered anions are NO_3_^−^, CO_3_^2−^, or SO_4_^2−^, which are accompanied by water molecules in the interlayer section [17,18]. The structure of an LDH consists of layers formed by the combination of trivalent and divalent cations in octahedral symmetry. Every metal cation is surrounded by several hydroxide anions and is connected to each other to create an extensive two-dimensional layer, which has a positive charge [18]. In the interlayer section, the anions stabilize the positive charges, and the water molecules create hydrogen bonds between the metal hydroxide layers. As a result, a structurally stable multilayered material is constructed [16,17,18].

Considering the distinctive nature of LDHs, their use has expanded to several fields, including photochemistry [19], drug delivery and health-related applications [20,21,22], catalysis [23], and as a sorbent material [24,25,26,27]. Although the sorption properties of LDHs enable their use in analytical chemistry, the limited number of free hydroxyl groups of the metal hydroxide layer significantly lower their sorption performance [28,29]. To overcome this limitation, many studies take advantage of these binding sites and the inherent positive charged environment of LDHs to fabricate complex three-dimensional nanocomposites with increased sorption abilities. Thus, LDHs have been modified with magnetic nanoparticles [30], graphene [31], and anionic surfactants [32], demonstrating increased extraction or removal capacities compared to plain LDH. Functionalizing an LDH with GQDs is a novel combination with many desirable properties. The GQDs have been used in combination with ZnAl LDH and MgAl LDH for the removal of cadmium [33] and organic dyes [29], respectively. The GQDs-LDH are rising nanocomposite materials with characteristics that can be used for new analytical applications. The lack of literature about the utilization of such materials on emerging and established pollutants calls for further research.

In this study, GQDs from citric acid were synthesized and incorporated into a MgAl LDH (Mg_6_Al_2_(OH)_16_∙4H_2_O, [34]). The synthesis of the LDH was optimized and modified so as to eliminate the presence of carbonate ions during the synthesis in an effort to increase the loading with GQDs. The composite material was used in a dispersive solid-phase extraction procedure for the extraction of representative compounds from three classes of compounds, i.e., benzophenones, phenols, and parabens. Phenols are known for their toxic effects on organisms and their persistence in the environment [35]. Benzophenones are commonly employed in many cosmetic products, and as such, they can easily be transferred to the aquatic environment, causing many adverse health effects [36]. Likewise, parabens extensively occur in daily use products, and due to their toxicity, they pose a threat. Due to the adverse effects of the above compounds, their detection is necessary and a long-lasting requirement for analytical methods should be fulfilled [37]. Based on the sample preparation procedure developed herein, an analytical method was proposed for the determination of six representative compounds in water samples. The compounds examined herein are among the most commonly used from each selected class [38,39].

## 2. Results and Discussion

### 2.1. Synthesis Optimization

Experiments were carried out in order to optimize the synthesis of the composite nanomaterial. The main criterion used was the total extraction efficiency (%) for the sum of the examined compounds. The quantification for each analyte was carried out at the wavelength where the UV absorbance was maximum, according to Table S1 of Supplementary Material.

#### 2.1.1. Carbonate-Containing and Carbonate-Free Mg-Al LDH

Most LDHs are produced following coprecipitation methods, under alkaline conditions. A potential drawback of this method is that the produced LDH contains carbonate ions in the interlayer space due to the dissolution of CO_2_ from air into the solution. The content of the LDH in carbonate ions makes it more difficult to incorporate other ions or molecules in the interlayer space because LDH and carbonate ions have an exceptionally high affinity [40]. To examine whether the presence of carbonate ions would make the functionalization of the LDH with CNDs more difficult, resulting in a less efficient material, the synthesis of the composite material was carried out in the presence and absence of carbon dioxide (under a continuous nitrogen flow). The two synthesized composites were used for the extraction of the tested compounds. The results were conclusive that the composite material containing carbonate ions achieved a 44% lower extraction efficiency compared to the carbonate-free analogue (results not shown).

#### 2.1.2. Different Types of GQDs

According to a previous study, the carbonization degree of citric acid results in the production of different products [41]. We examined whether using the produced GQDs after 30 min or after 120 min of heating citric acid is more appropriate to produce a composite material with a better extraction performance. In this context, citric acid was heated for 30 and 120 min, and the products were used for the synthesis of the carbonate-free LDH/GQDs. According to the results, the use of the GQDs heated for 30 min resulted in a composite with a better performance (~20%) compared to those heated for 120 min. According to Dong et al., GQDs after 30 min of carbonization have a typical size of 15 nm, while after 120 min the size is increased [41]. Therefore, our results can be attributed to the smaller size of the 30 min GQDs compared to the 120 min GQDs, resulting in a higher surface area, as well as the better intercalation in the interlayer space of the LDH [41]. Therefore, QGDs after 30 min of carbonization were used for further experiments.

#### 2.1.3. Amount of GQDs

To maximize the extraction efficiency, the loading of the LDH with GQDs was examined (using 0.5, 1.0, 1.5, and 2.0 mL of a GQDs solution of 20% *w*/*v*). According to the results, the use of 1.0 mL of the GQDs solution improved the extraction efficiency of the composite by 18%, compared with the composite containing the 0.5 mL GQDs solution. Further improvement was not achieved using higher volumes of the GQDs solution. Therefore, the use of 1.0 mL of the GQDs solution was deemed necessary to incorporate the maximum amount of GQDs into the LDH and to achieve an optimum performance.

To explore the role of every single component of the material, the extraction of the target compounds was carried out using plain LDH. It was revealed that the plain LDH exhibited almost 80% lower extraction efficiency for benzophenones and almost 70% lower for phenols and parabens. Then, we examined the repeatability and inter-day repeatability: five batches of the material were synthesized on the same day and on five consecutive days. The synthesized materials were tested for their extraction efficiencies. The results were expressed as the relative standard deviation (RSD) of the different batches. The repeatability of the synthesis was between 2.1 and 3.2%, and the inter-day repeatability was between 2.6 and 4.1%, for all target compounds.

### 2.2. Material Characterization

**Figure 1:**
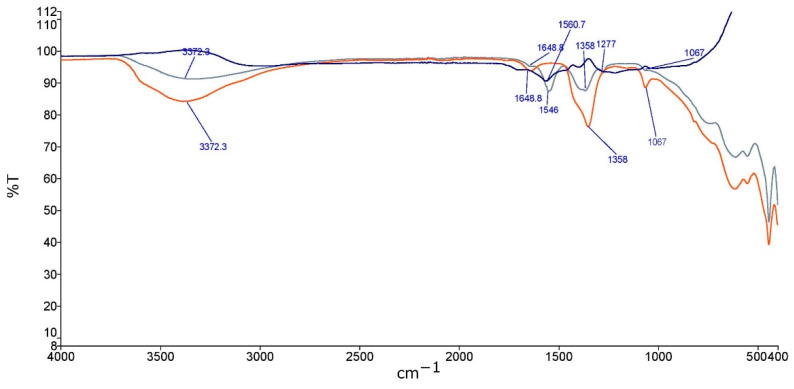
FTIR spectra of bare LDH (red spectrum), GQDs (blue spectrum), and LDH/GQDs composite (green spectrum).

Figure 1 depicts the FTIR spectrum of the bare LDH, the CQDs, and the composite material. A broad absorption band appears at 3385 cm^−1^ which can be attributed to the stretching vibrations of the -OH groups [42]. The peak centered at 1353 cm^−1^ is due to the asymmetric stretching vibrations of the intercalated NO_3_^−^, the peak at 1640 cm^−1^ is due to the flexural oscillation peaks of the interlayer water molecules, and the peak at 1067 cm^−1^ is due to the Al-OH stretching [43]. The FTIR spectrum of the GQDs shows a peak at 1560 cm^−1^, which is due to the asymmetric stretching of the -COO^−^ group. Two small shoulders at around 1400 and 1650 cm^−1^ can be attributed to the symmetric stretching of the -COO^−^ group and the vibrations of the -C=O groups, respectively [41]. Finally, the spectrum of the GQDs-LDH composite material exhibits a broad peak at around 3353 cm^−1^, due to the stretching of the -OH groups. Moreover, a peak at 1548 cm^−1^ can be seen, which is due to the asymmetric stretching of the -COO^−^ groups of the GQDs. The peak at 1358 cm^−1^ is shorter compared to the FTIR spectrum of the bare LDH, hinting toward the displacement of the intercalated NO_3_^−^ by the GQDs. Finally, the peaks at 1277 and 752 cm^−1^ are due to the Al-OH stretching vibrations [43].

**Figure 2:**
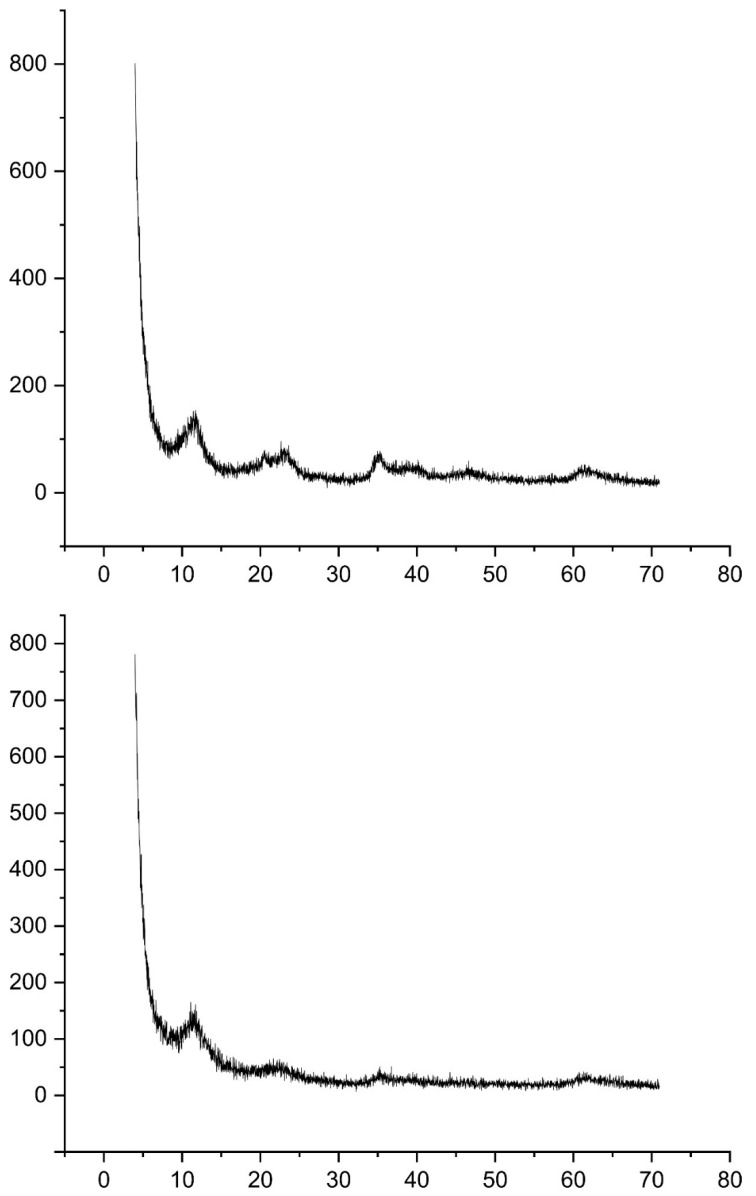
XRD spectra of bare LDH (**upper spectrum**) and the composite material (**lower spectrum**).

**Figure 3:**
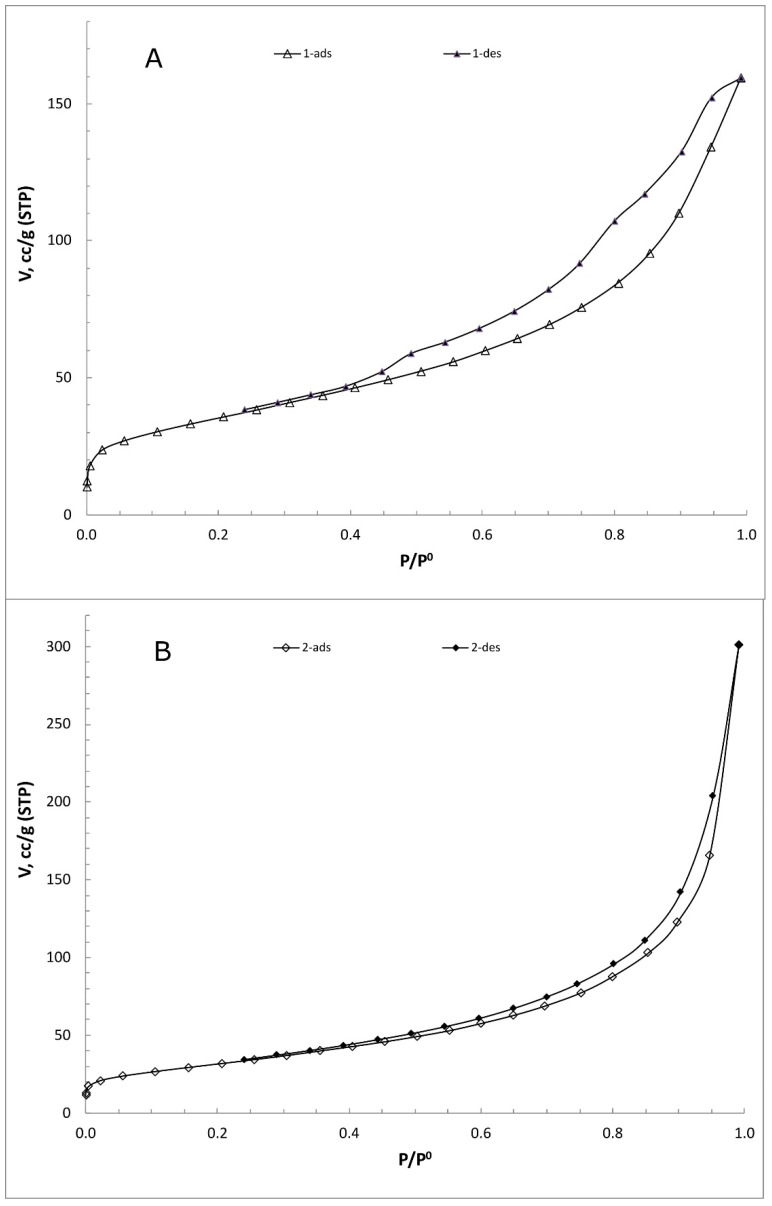
Nitrogen adsorption-desorption isotherms of (**A**) bare LDH and (**B**) LDH/GQDs composite.

In Figure 2, the XRD spectra of the bare LDH and the composite material can be seen. Diffraction peaks at 11°, 22°, and 35° can be seen, which correspond to the (003), (006), and (009) planes of the Mg-Al LDH, suggesting a crystallized hydrotalcite structure [44,45]. In the XRD spectrum of the composite material, a broader peak at around 20^o^ can be seen, suggesting the addition of the GQDs in the structure of the LDH [46]. In order to further validate the addition of the GQDs in the LDH structure, during the synthesis of the composite material, the surface areas of the bare LDH and the composite material were measured. The surface of the bare LDH was calculated as 126.1 m^2^ g^−1^, and that of the composite material was 114.7 m^2^ g^−1^. The difference in the surface area is due to the addition of the GQDs in the structure of the LDH (Figure 3).

### 2.3. Optimization of the Extraction Procedure

In order to maximize the extraction efficiency of the proposed method, experiments were carried out to optimize the parameters of the extraction (i.e., the sample pH, ionic strength, temperature, stirring time and stirring rate, sample volume, and the amount of sorbent) by using 10 mL double-distilled water (DDW) spiked with 200 μg L^−1^ of each analyte, and 10 mg of the sorbent was added. A one-step-at-a-time approach was employed, resulting in a stepwise increase in the total extraction efficiency (%) with each optimized parameter, resulting in a 100% extraction efficiency.

#### 2.3.1. pH and Mechanism of Interaction

**Figure 4:**
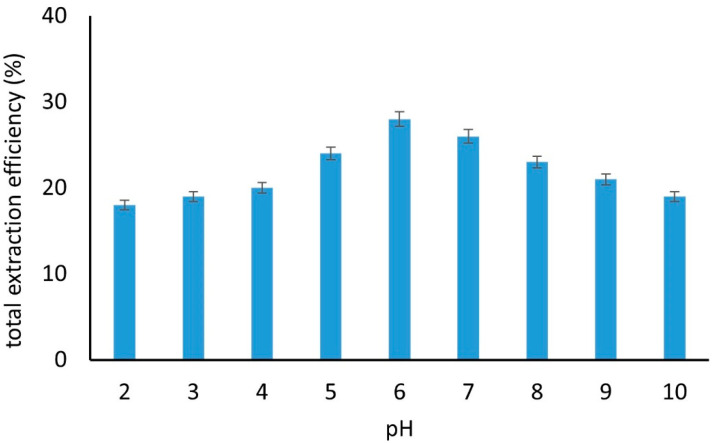
Total extraction efficiency of the examined compounds from solutions with different pH using the GQDs-LDH composite material (number of replicate analyses = 3).

As can be seen in Figure 4, the maximum total extraction efficiency was achieved when the pH was adjusted to 6.0. The lower extraction efficiencies achieved in more alkaline solutions were mainly due to the parabens (the extraction efficiency at pH 10 was ~5%). This can be justified by the hydrolysis of parabens that takes place in acidic or alkaline conditions [47]. Ιn more acidic conditions, the reduction in the extraction efficiency was more pronounced compared to the alkaline conditions, implying that this efficiency was not attributed solely to the hydrolysis of the parabens. According to a previous study, the dissolution of the MgAl LDH material takes place at an acidic ambiance (pH < 4) [48]. To further test whether this is the case, a suspension of the composite material in the DDW (pH 4.0) was prepared, and after stirring for 1 h, the fluorescence of the solution was recorded (if the dissolution of Al^3+^ and Mg^2+^ takes place, then fluorescent GQDs are released into the solution). The results showed that the solution exhibited a weak fluorescence. The same experiment was repeated using a solution at pH = 6.0. The emitted fluorescence was 80% lower than that at pH 4.0. When 30 min of stirring was employed instead of 1 h, no fluorescence emission was recorded. Hence, no dissolution of the composite material was recorded at pH 6, after 30 min. As a result, the pH 6.0 was selected as the optimum condition for the extraction of the compounds. Based on the sorption profile of the tested compounds at various pH, it can be inferred that the compounds are extracted, mainly, via hydrophobic interactions (as can be seen in Table S1, all the compounds have log*K*_ow_ values in the range of 3.0–4.3).

#### 2.3.2. Ionic Strength

**Figure 5:**
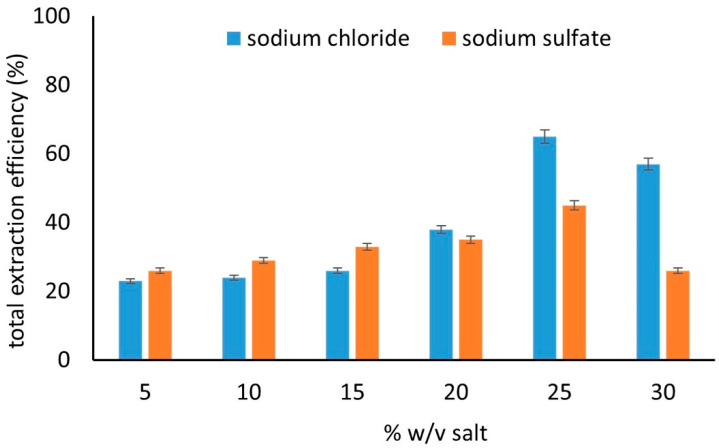
Total extraction efficiency of the examined compounds from solutions with different ionic strength using the GQDs-LDH composite material (number of replicate analyses = 3).

We tested the extraction efficiency of the synthesized composite material using a solution containing sodium chloride or sodium sulfate (5–30% *w*/*v*). The results are depicted in Figure 5. Generally, the addition of salt is beneficial for the extraction of the compounds. This can be attributed to the salting-out effect, which lowers the solubility of organic compounds in aqueous solutions and favors their extraction [49]. When sodium chloride was used, the optimum performance was achieved when 25% *w*/*v* of the salt was used. A higher percentage increases the viscosity of the solution, thus lowering the diffusion rate [49]. When sodium sulfate was used, the total extraction yield was also increased, albeit to a lower degree compared to the sodium chloride. This can be justified by the low colloidal stability of MgAl LDH in the presence of different ions [50]. According to a previous report, the colloidal stability of the LDH is affected by the presence of ions with different valences. Between chloride and sulfate ions, the latter renders the LDH more unstable and thus aggregates are formed more readily [50]. This can significantly decrease the performance of the sorbent material. Therefore, the use of 25% *w*/*v* sodium chloride was selected for further experiments.

#### 2.3.3. Temperature of Extraction

**Figure 6:**
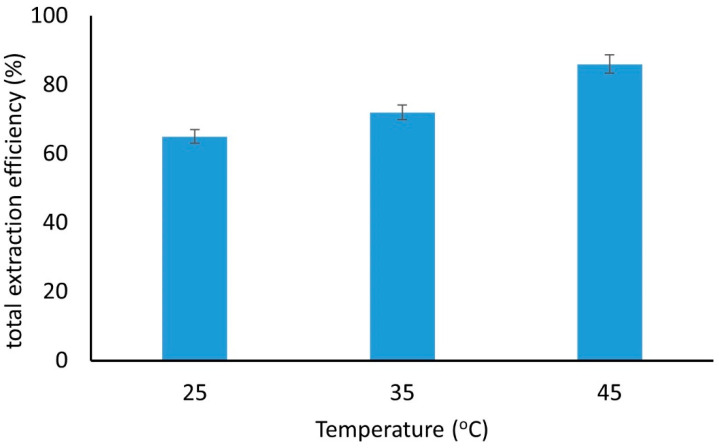
Total extraction efficiency of the examined compounds at three temperatures using the GQDs-LDH composite material (number of replicate analyses = 3).

The extraction experiments were carried out at three temperatures, i.e., 25, 35, and 45 °C, using the optimum parameters, as established before (no pH adjustment and addition of 25% *w*/*v* NaCl), and the results are given in Figure 6. As can be seen, as the temperature increases, the total extraction efficiency increases too. An increase in the temperature by 20 °C causes about a 20% increase in the extraction efficiency. This can be justified by two main reasons: First, the mechanism of interaction between the sorbent and the examined compounds was probably due to hydrophobic interactions, as mentioned in Section 2.3.1. It is known that as the temperature increases, hydrophobic interactions are favored [51]. Secondly, the high viscosity due to the high salt content is lowered as the temperature increases and this counterbalances the negative impact of the high viscosity of the solution on the extraction performance. Therefore, further experiments were carried out by heating the solution at 45 °C before and during the extraction step.

#### 2.3.4. Other Extraction Parameters

**Figure 7:**
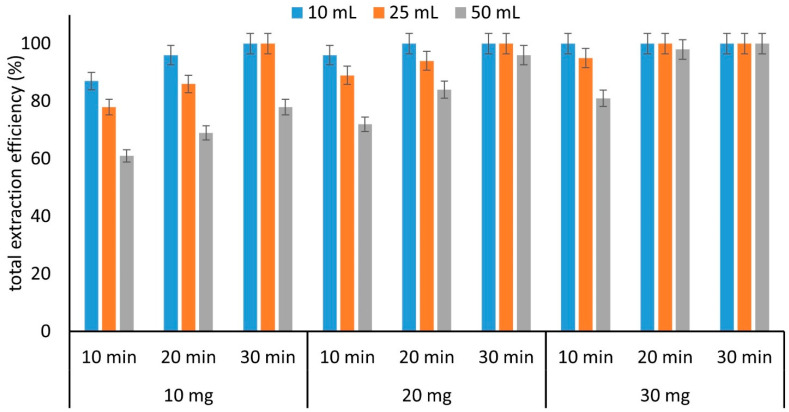
Total extraction efficiency of the examined compounds with respect to sample volume, amount of sorbent (GQDs-LDH), and extraction time (number of replicate analyses = 3).

Other parameters that affect the extraction performance of the material are the stirring time and stirring rate, sample volume, and the amount of sorbent. However, these parameters are interrelated, and certain combinations of their values may be critical. Preliminary experiments were carried out regarding the stirring rate during the extraction, examining rates of 500, 700, and 900 rpm. The results were conclusive that a high stirring rate favored the extraction process. This was anticipated because the ionic strength of the solution was high and could lower the mass transfer during the extraction process. Therefore, for further experiments, a high stirring rate (900 rpm) was used. To assess the rest of the parameters, the experiments were carried out using 10, 20, and 30 mg of sorbent, three sample volumes (10, 25, and 50 mL, containing the same concentration of analytes) at three time intervals (10, 20, and 30 min), resulting in a total of 27 experiments. The results can be seen in Figure 7. The optimum performance can be achieved for various combinations of the three examined parameters. For the method development, we selected the use of 10 mg of sorbent material (for a reduced consumption and thus, lower cost of analysis) for 25 mL of sample (for increased enrichment values) and a stirring time of 30 min.

Under optimum conditions, the total extraction efficiency of the GQDs-LDH composite was examined and compared to that of the bare LDH. From the results, it was apparent that the bare LDH could achieve ~20% extraction of the examined compounds, while the composite material could extract 100% of the compounds. Therefore, it can be inferred that the LDH serves a dual role in the composite material: Firstly, it can assist in the overall extraction process, albeit to a low extent, and secondly, it serves as a good supporting material for the GQDs, making it feasible for them to be used in a sample preparation procedure because they cannot be used bare.

### 2.4. Optimization of Elution Conditions and Reuse of the Material

To desorb the analytes from the sorbent material, acetonitrile and methanol were tested. When 1 mL of each solvent was used (1 mL solvent was added to the material and the mixture was placed in an ultrasonic bath for 30 s), both solvents could desorb nearly 70% of all the analytes, while a second elution step desorbed the remaining 30% of the analytes. For further experiments, methanol was selected. Next, we examined whether the addition of 2 mL of solvent in a single step could achieve the same results as the addition of 2 × 1 mL of the solvent. It was proven that the addition of 2 mL of solvent in a single step was adequate to fully desorb the analytes.

Next, we evaluated whether the material could be reused after the elution step. Following a second extraction and elution step, the recoveries of the analytes were almost the same as the first-time extraction (<3% decrease in the total extraction efficiency). After a third extraction step, the recoveries of the analytes slightly decreased compared to the first-time extraction (~7% decrease in the total extraction efficiency). Therefore, the material can be reused for more than one extraction–elution cycle, resulting in a method with a lower cost.

### 2.5. Analytical Figures of Merit

**Figure 8:**
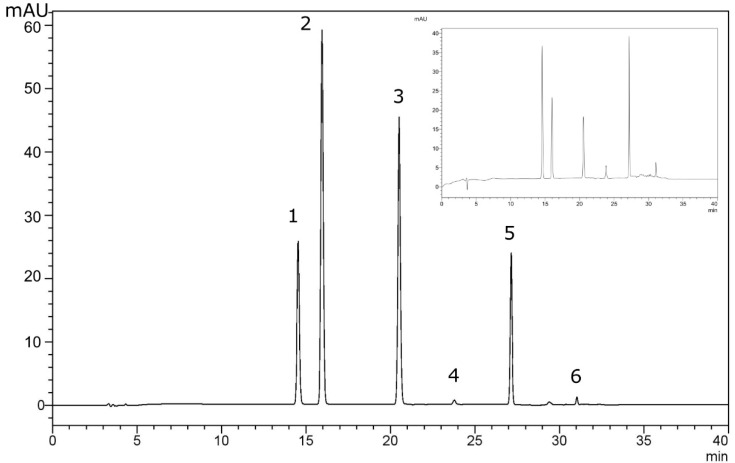
Chromatogram of the extract obtained from a spiked lake water sample (10 μg L^−1^) with diode array detector (254 nm) using the proposed procedure; 1: 4OH-BP; 2: PPB; 3: BPB; 4: TCP; 5: BP-3; 6: OCP. The discrepancies of peak heights are attributed to the different molecular absorption maxima of analytes. Inset: the chromatogram at 286 nm.

Under the optimum conditions, an analytical method was developed for the simultaneous determination of the three classes of compounds. The compounds are representative of each class and the method can be used for other compounds of the same class, as well. The limit of quantification (LOQ) for each compound was determined after analyzing extracted samples containing the compounds, at a signal-to-noise ratio of 10. The LOQs were found to be between 0.10 and 0.17 μg L^−1^ for the benzophenones and parabens, while for the phenols, they were 1.28 and 1.33 μg L^−1^, respectively. Calibration curves were prepared, with the lowest concentration of each compound being the LOQ and the highest the 200 μg L^−1^. The linear equations, as well as the rest of the analytical figures of merit of the developed procedure, are given in Table 1. The coefficients of determination were between 0.9980 and 0.9987, suggesting a good linearity in the examined range. Next, the enrichment factors were calculated according to our previous reports [52,53]. They were found to be between 170 and 186 for all the target analytes. Next, we evaluated the precision of the method. The repeatability was studied by analyzing five samples within the same day, and the inter-day repeatability was assessed by analyzing three samples each day for three consecutive days. The results, expressed as RSD, are given in Table 2. As can be seen, the repeatability of the method was between 5.0 and 6.9% and the inter-day repeatability was between 6.7 and 8.2%. Finally, the relative recoveries of the method were calculated by analyzing water from the lake Pamvotis (Ioannina, Greece) spiked with concentrations corresponding to 2 times and 10 times of LOQ, for each compound. A representative chromatogram is given in Figure 8. The recoveries were found in the range of 92–99% for the lowest tested concentration and 94–100% for the highest tested concentrations. Overall, all the above figures of merit suggest that the developed method is suitable for the reliable analysis of the examined compounds.

## 3. Materials and Methods

### 3.1. Chemicals and Reagents

4-Hydroxybenzophenone (4OH-BP) (>98%), 2-hydroxy-4-methoxybenzophenone (BP-3) (98%), 2,3,5-trichlorophenol (TCP), 4-octylphenol (OCP) (99%), propyl para-hydroxybenzoate (PPB), butyl 4-hydroxybenzoate (BPB), sodium chloride, sodium sulfate, hydrochloric acid (37% *w*/*w*), ammonium hydroxide (28% *w*/*w*), citric acid (≥99.5%), sodium hydroxide (≥97%, pellets), magnesium nitrate hexahydrate (Mg(NO_3_)_2_·6H_2_O, 99%), aluminum nitrate nonahydrate (Al(NO_3_)_3_·9H_2_O, ≥98%), formic acid (96%), and all solvents (HPLC-grade) were purchased from Sigma-Aldrich (Hellas, Greece). Stock standard solutions of each analyte (2000 mg∙L^−1^) were prepared in acetonitrile. Double-distilled water was used throughout the experiments

### 3.2. Instrumentation

The FTIR spectra of the materials were recorded on a Spectrum Two FTIR using an attenuated total reflectance accessory (PerkinElmer, MA, USA). A Shimadzu (Kyoto, Japan) HPLC system, consisting of a DGU-20A3R online degassing unit, two LC20AD pumps, a SIL-20AC HT autosampler, a CTO 20AC column oven, and an SPD-M20A Diode Array Detector, was used for separation and analysis of the samples. A Hypersil Gold (250 mm × 4.6 mm, 5 μm particle size) column from Thermo Fisher Scientific (San Jose, CA, USA) was used for the separation. The column was placed in an oven at 30 °C. The mobile phase consisted of water (A) and acetonitrile (B), both containing 0.1% (*v*/*v*) formic acid. For the separation of the analytes, the following gradient program was employed: the concentration of B was increased from 5 to 95% in 40 min. The flow rate of the mobile phase was 1.0 mL/min. Flow rate and temperature were selected so as to avoid increased system temperatures. The detector was set at a wavelength range of 200–360 nm. Peak identification was achieved by comparing the retention times and UV spectra of peaks in samples with those of pure compounds. The specific surface areas were calculated based on nitrogen adsorption-desorption porosimetry according to the BET method.

### 3.3. Graphene Quantum Dots (GQDs) Synthesis

The synthesis of GQDs was carried out according to a previous report [41,54]. In brief, 0.3 g of citric acid was added to a test tube and heated at 200 °C for 30 min in an oil bath. After cooling to room temperature, 5 mL of water was added and the mixture was vortexed until the viscous orange-yellow liquid was dissolved. The GQD solutions were stored at 4 °C.

### 3.4. Synthesis of Carbonate-Free Mg-Al LDH and Mg-Al LDH-Incorporating GQDs

The synthesis of the LDH/GQD composite, that is, free from carbonates as interlayer anions, was based on a previously reported method [34]. Two aqueous solutions were prepared: one containing 0.80 mol·L^−1^ of Mg(NO_3_)_2_·6H_2_O and 0.40 mol·L^−1^ of Al(NO_3_)_3_·9H_2_O and another one with 1.67 mol L^−1^ of NaOH. In a glass beaker, 1 mL of GQDs solution (20% *w*/*v*) was added, and degassing was carried out using a constant nitrogen flow. Then, 2 mL of each of the abovementioned solutions were added dropwise, simultaneously, under vigorous stirring and nitrogen flow. Then, the solution was stirred at 65 °C for 4 h under nitrogen flow, and the mixture was centrifuged at 3000 rpm for 5 min. The precipitate was rinsed several times with water and ethanol and placed in an oven at 60 °C overnight. The bare LDH material was prepared by using 1 mL of water in the first step of the synthesis, instead of GQDs solution.

### 3.5. Optimized Extraction Procedure

In a glass beaker, 25 mL of sample (whose pH was adjusted to 6.0) and NaCl (25% *w*/*v*) were added. After dissolving the salt with stirring, the solution was heated at 45 °C. Next, 10 mg of the sorbent was added, and the mixture was stirred for an additional 30 min at 900 rpm. The sorbent was isolated by centrifugation at 3500 rpm, for 5 min. The supernatant was decanted, and the sorbent was washed with DDW. After decanting the water, 2 mL of acetonitrile was added, and the mixture was sonicated for 30 s. The solvent was collected and evaporated to dryness under a gentle nitrogen stream. Finally, the residue was reconstituted in 100 μL of the mobile phase and injected into an HPLC system. For the analysis of lake water, the sample was filtrated through a Whatman filter to remove particulate matter, and then the pH was adjusted to 6.0 with hydrochloric acid.

## 4. Conclusions

In this study, the use of an LDH-GQDs composite material as a sorbent is described for the first time, for the extraction of three classes of compounds. The LDH serves as a solid support material for the GQDs and contributes to the overall extraction procedure. In this way, the GQDs can be used in a sample preparation procedure. The developed composite material was utilized in a dispersive solid-phase extraction procedure and the method exhibits good analytical figures of merit. Overall, the described procedure can be used as an alternative to or a substitute for the existing methods for the determination of the target compounds, and it paves the way for the development of more advanced sorbent materials that utilize GQDs for sample preparation.

## Figures and Tables

**Table 1:** Analytical figures of merit of the developed analytical method.

Compound	Linear Equation	Coefficient of Determination, *R*^2^	LOQ (μg L^−1^)	Enrichment Factor
4OH-BP	Y = 20,986 + 18,745	0.9987	0.13	178
PPB	Y = 28,206x + 31,685	0.9986	0.10	175
BPB	Y = 22,380x + 15,503	0.9980	0.12	186
TCP	Y = 2582.3x + 1150.9	0.9983	1.33	169
BP-3	Y = 16,578x + 15,009	0.9984	0.17	179
OCP	Y = 1696.6x + 1509.9	0.9985	1.28	170

**Table 2:** Relative standard deviations and relative recoveries of the examined analytes from lake water.

Analyte	RSD (%)		Relative Recovery (%)	
	Repeatability (*n* = 5)	Inter-Day Repeatability (*n* = 3 × 3)	2 × LOQ	10 × LOQ
4OH-BP	5.0	6.7	93	94
PPB	5.5	7.9	99	100
BPB	5.9	8.0	92	93
TCP	5.6	7.8	92	96
BP-3	6.4	7.3	94	95
OCP	6.9	8.2	94	98

## Data Availability

The data presented in this study are available on request from the corresponding author.

## Supplementary Materials

The following supporting information can be downloaded at: https://www.mdpi.com/article/10.3390/molecules27238388/s1, Table S1: Chemical structure, physicochemical characteristics, retention time in the HPLC system, and maximum UV absorbance of the examined compounds.

## Sample Availability

Samples of the compounds are not available from the authors.
