# Supplementary material for: Layered Double Hydroxide/Graphene Quantum Dots as a New Sorbent for the Dispersive Solid-Phase Microextraction of Selected Benzophenones, Phenols, and Parabens
Source: Molecules. 2022 Dec 1;27(23):8388. doi: 10.3390/molecules27238388 (PMC9738325; doi:10.3390/molecules27238388)
Supplement: Supplementary file 1 [file molecules-27-08388-s001.zip › molecules-2008668-supplementary.pdf]

Supplementary Material

Layered double hydroxide/graphene quantum dots as a new sorbent for the dispersive solid-phase microextraction of selected benzophenones, phenols, and parabens

Theodoros Chatzimitakos *, Alkiviadis Vasilas and Constantine Stalikas

Laboratory of Analytical Chemistry, Department of Chemistry, University of Ioannina, 45110, Ioannina, Greece

* Correspondence: t.chatzimitakos@uoi.gr; Tel.: 00302651008725

Table S1. Chemical structure, physicochemical characteristics, retention time in the HPLC system, and maximum UV absorbance of the examined compounds.

Compounds	Chemical Structure	LogKow	Pka	Retention time (min)	Maximum UV absorbance (nm)
4-hydroxybenzophenone (4OH-BP)	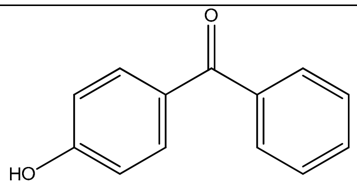	3.07	8.14	14.6	291
propyl parahydroxybenzoate (PPB)	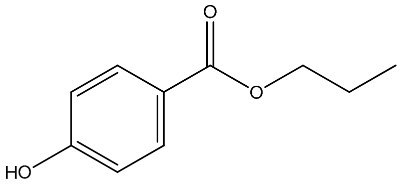	3.04	8.50	16.1	254
butyl 4-hydroxybenzoate (BPB)	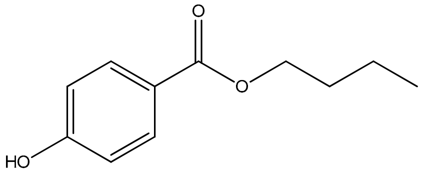	3.57	8.47	20.6	254
2,3,5-trichlorophenol (TCP)	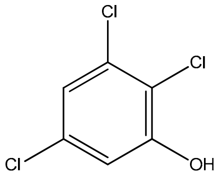	3.84	7.00	23.9	286

2-hydroxy-4-methoxybenzophenone (BP-3)	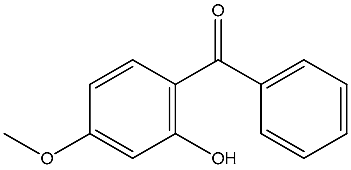	3.64	7.56	27.2	286
4-octylphenol (OCP)	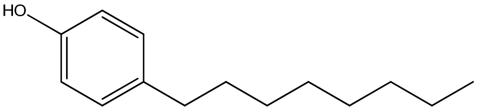	4.30	10.15	31.1	276
